# Morphological and physiological adaptive profiles of Spanish and Brazilian goat breeds in arid environments

**DOI:** 10.1007/s11250-026-05007-8

**Published:** 2026-04-01

**Authors:** Wallace Sostene Tavares da Silva, Robson Mateus Freitas Silveira, Jacinara Hody Gurgel Morais Leite, Luis Alberto Bermejo, Alexandr Torres Krupij, Débora Andréa Evangelista Façanha

**Affiliations:** 1 Department of Animal Science, Federal Rural University of the Semi-Arid Region (UFERSA), Mossoró, RN 59625-900 Brazil; 2 Department of Animal Science, “Luiz de Queiroz” Agriculture College (ESALQ), University of São Paulo (USP), Piracicaba, São Paulo State 13418-900 Brazil; 3 Department of Animal Science, Federal University of Paraíba (UFPB), Areia, PB Brazil; 4 Departamento de Ingeniería Agraria y del Medio Natural, Escuela Politécnica Superior de Ingeniería – Sección Agraria, Universidad de La Laguna (ULL), Carretera de Geneto nº2, San Cristóbal de La Laguna (Tenerife), 38200 Spain; 5 Animal Production Unit, Pastures and Forages in Arid and Subtropical Zones, Canarian Institute of Agricultural Research (ICIA), PBOX n° 60, La Laguna, Santa Cruz de Tenerife, 38200 Spain; 6 University of International Integration of Afro-Brazilian Lusophony (UNILAB), Redenção, CE 62790-790 Brazil

**Keywords:** Brazilian semi-arid, Canary Islands, Coat traits, Heat tolerance, Physiological response

## Abstract

Goats living in arid and semi-arid environments combine morphological and physiological strategies to maintain homeothermy; autochthonous breeds from the Canary Islands and Brazil’s semi-arid region offer a natural model to compare these adaptive profiles. In this study, we characterized the adaptive profile of five populations: Majorera, Palmera, and Northern Tinerfeña (Spain) and Canindé and Moxotó (Brazil). A total of 150 goats (30 per breed), all clinically healthy, non-pregnant, non-lactating females of reproductive age (2–4 years), were evaluated. Morphological traits included coat thickness (CT), hair diameter (HD), and hair length (HL); physiological indicators included rectal temperature (RT) and respiratory rate (RR). Among the Spanish breeds, RT did not differ (*p* > 0.05; ≈ 39.2 °C), whereas RR was higher in North Tinerfeña (*p* < 0.05) than in Majorera and Palmera. Palmera and North Tinerfeña showed greater CT and HL (*p* < 0.05; CT) compared with Majorera (CT ≈ 1.06 mm; HL ≈ 36.7 mm), and HD also differed among the Spanish breeds (*p* < 0.05). In the Brazilian breeds, RT did not differ (*p* > 0.05); however, Canindé exhibited higher RR (*p* < 0.05), CT (*p* < 0.05), and HL (*p* < 0.05), while HD was similar between breeds (*p* > 0.05). In multivariate analyses, principal component biplots indicated predominantly morphological variability in the Spanish breeds and predominantly physiological variability in the Brazilian breeds; correspondence analysis clearly separated Spanish and Brazilian groups; discriminant analysis confirmed this distinction (Wilks’ λ, *P* < 0.001) and achieved higher classification accuracy when morphological variables were considered, especially for classifying Majorera. On their own, physiological variables had lower discriminatory power in the Brazilian breeds. Interestingly, Majorera clustered closer to Moxotó and Canindé in multivariate space, while remaining distinct from Palmera and Northern Tinerfeña. Collectively, the results reveal two complementary adaptive profiles: (i) morphological insulation that attenuates heat gain and reduces demand for active heat loss (predominant in Spanish breeds), and (ii) physiological adjustment emphasizing evaporative heat loss (predominant in Brazilian breeds). These findings provide actionable evidence for genetic resource conservation and inform breeding programs oriented toward thermal resilience under warming and aridification scenarios.

## Introduction

Goat farming is strategic for production systems in arid and semi-arid environments, and in the Canary Islands it forms a unique historical–biogeographical mosaic. Three officially recognized autochthonous breeds, Majorera (prevalent in Fuerteventura/Gran Canaria/Lanzarote), Palmera (La Palma), and Tinerfeña (Tenerife), total more than 200,000 animals and support high value-added dairy chains (Silveira et al. 2024; Senczuk et al. 2024). Recent genomic and ecological evidence indicates that intra-archipelago differentiation mirrors marked environmental gradients and local adaptation processes, including the coexistence of ecotypes in northern (more humid) and southern (drier) Tenerife, making these breeds a natural model for investigating heat tolerance (Senczuk et al. 2024).

In parallel, in Brazil’s northeastern semi-arid region, native breeds such as Canindé and Moxotó have been historically selected under high thermal loads, intense radiation, and hydric seasonality, contributing to the resilience of extensive systems and to regional food security (Gomes et al. 2008; Façanha et al. 2020; Ferreira et al. 2020). Comparing the Canarian context, which ranges from the aridity of Fuerteventura/Lanzarote to the more humid conditions of La Palma and northern Tenerife, with the Brazilian semi-arid offers a contrasting environmental gradient that is ideal for phenotypic and functional investigation.

In small ruminants, the response to heat stress results from the integration of physiological, morphological, behavioral, and genetic mechanisms: increased respiratory rate and evaporative heat loss; adjustments in water balance; use of shade and microclimates; and variations in coat architecture and color that modulate the absorption or reflectance of radiation and convective exchanges (Sejian et al. 2018; Lima et al. 2022).

Studies show that longer and denser coats, especially with fibers of greater diameter, attenuate radiative heat gain, reducing rises in body temperature and the ventilatory demand under direct sunlight, suggesting a “morphological insulation” profile (Silveira et al. 2024; da Silva et al. 2025a, b). Conversely, in goats adapted to the tropics, a short and sleek coat favors convective dissipation but entails greater reliance on evaporative mechanisms (panting or sweating), characterizing a “physiological adjustment” profile (Façanha et al. 2023; Silveira et al. 2024, 2026; da Silva et al. 2025a, b ab). In light of this framework, we hypothesize that the Canarian breeds tend to predominantly express the morphological insulation profile, consistent with environments of high radiant load and trade winds that help maintain a superficial thermal gradient, whereas the Brazilian breeds Canindé and Moxotó would represent a physiological adjustment profile, compatible with persistent thermal stress and lower water availability.

This study aims to: (i) determine the adaptive profile, physiological and morphological, of five local goat populations from Spain (Canary Islands) and Brazil (northeastern semiarid); and (ii) identify which coat and physiological response variables have the greatest discriminative power for classifying the adaptive profile among these populations, providing evidence for conservation and for breeding programs oriented toward heat tolerance.

## Materials and methods

### Site and period — Spain experiment

The study was conducted at the Instituto Canario de Investigaciones Agrarias (ICIA), an autonomous body of the Canary Islands regional government (Tenerife, Spain). Tenerife lies at 28°19′ N, 16°34′ W, is the central and largest island of the archipelago, with predominantly volcanic soils. The climate is Mediterranean-like, with low annual thermal amplitude (~ 18–25 °C) and moderate to low rainfall (~ 400–1000 mm·yr⁻¹). Under the Köppen system the island exhibits a Mediterranean type with little thermal seasonality. Although in the subtropics and close to Africa, conditions are moderated by northeasterly trade winds linked to the Azores High. Vegetation is altitudinally stratified, from succulent communities (~ 50 m a.s.l.) to high-elevation formations above ~ 1500 m.

### Animals — Spain experiment

Ninety adult goats—30 Majorera, 30 Palmera, and 30 North Tinerfeña—all female, of reproductive age (2–4 years), non-pregnant, clinically healthy, and non-lactating were evaluated. The breed descriptions are presented below: *Majorera*: dairy breed adapted to arid zones; short hair, polychromatic coat, sub-hypermorphic build; head profile straight to sub-convex, broad ears, horns common; udder typically dark and well developed; *Palmera*: adapted to montane areas with mild and humid climate; high dairy potential for value-added products; medium size, short neck, predominantly reddish coat with medium hair; head profile straight to sub-concave, short ears, well-developed horns; udder comparatively more compact; and *North Tinerfeña*: sub-hypermorphic, long-lined dairy type; mean withers height ~ 79.5 cm (males) and 70.2 cm (females); large, elongated head, straight to sub-convex profile; large, pendulous ears; prisca-type horns (born parallel, then diverging); frequent beard, forelock and skin appendages; predominantly black and brown coat with long hair; does show globose, dark-pigmented udders—traits linked to adaptation to the cooler, wetter north of Tenerife.

The production system adopted was intensive, in which the animals were stabled in sheds covered with metal plates, open on the sides, with a ceiling height of 4 m and a concrete floor. The walls of the stalls were 1 m high, with metal troughs arranged randomly, with no criterion other than breed for separating the animals. The animals were fed in the early hours of the morning (6:00 a.m.) when they received a single pelletized commercial feed as recommended by the INRA (2007), after which a chopped roughage was made available to the animals directly in the trough at the end of the day (2:00 p.m.: 00 min). The bulk was generally composed of native vegetation such as: moringa (*Moringa oleifera*), tagasaste (*Cytisus proliferus*), common canary tedera (*Bituminaria bituminosa*), vinegarra (*Rumex lunaria*), cornical (*Periploca angustifólia*), maralfafa (*Pennisetum purpureum*). Access to fresh water was *ad libitum*.

### Site and period — Brazil experiment

The study took place on a commercial farm in Lajes (Angicos microregion), Rio Grande do Norte, Brazil. The regional climate is semi-arid (Köppen), photoperiod varies little annually. Management was semi-intensive, based on native rangeland of hyperxerophilous Caatinga (dry vegetation with abundant cacti and low, sparse shrubs), with concentrate supplementation part of the day. Repeated measurements were collected from the same animals in March and September, during morning and afternoon sessions, including thermoregulatory and morphology.

### Animals — Brazil experiment

Sixty goats—30 Canindé and 30 Moxotó—all female, of reproductive age (2–4 years), non-pregnant, clinically healthy, and non-lactating were evaluated. The breed descriptions are presented below: . *Canindé*: medium, conical, elongated head; straight to sub-concave profile; short, erect spear-like ears; symmetric horns (upward, backward, outward; stronger/wider in males); black/brown eyes; well-conformed trunk; broad chest; straight, broad loin; medium, slightly sloped croup; strong, correct limbs; predominantly black coat with light patches (white/cream/brown); and *Moxotó*: small-framed; medium, conical, elongated head; sub-concave profile; small, erect ears; straight horns (males up/back/out; females up/back); dark, strong hooves; white or bay coat with a black stripe from horn base to muzzle (often encircling the eyes) and a black dorsal stripe over > 50% of the back; black triangle on the nape; black belly, udder and distal limbs (with occasional small white spots); short, bright hair; black skin and dark mucosae.

**Fig. 1:**
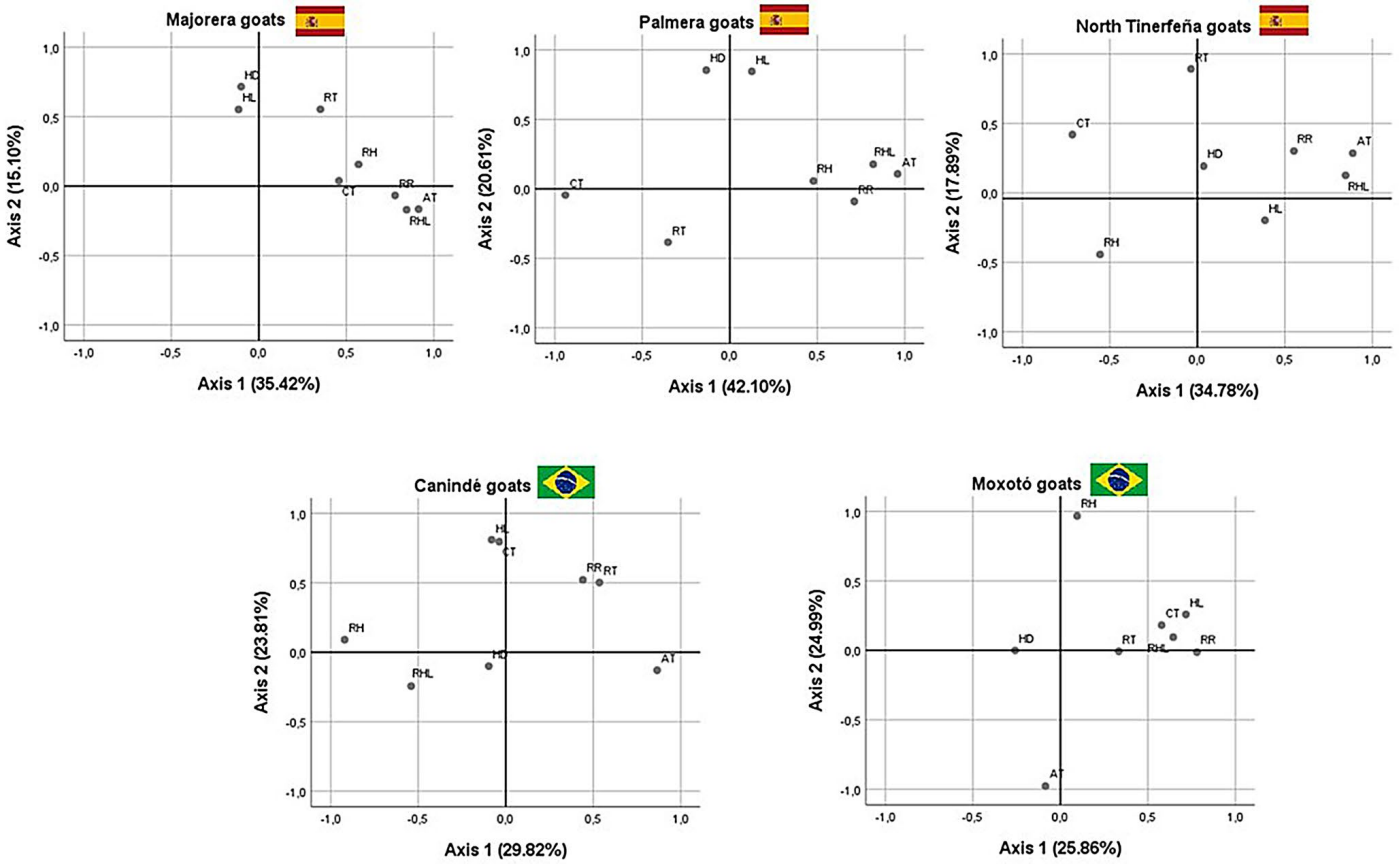
Principal component analysis (PCA) biplots by goat breed (Majorera, Palmera, North Tinerfeña, Canindé, and Moxotó), showing the contribution and association among physiological indicators, coat traits, and environmental variables. The percentages on the axes correspond to the variance explained by each component. RT - rectal temperature (°C); RR - respiratory rate (breaths/min); CT - coat thickness (mm); HL - hair length (mm); HD - hair diameter (µm), AT - air temperature (°C), RH – relative humidity (%), RHL, -radiant heat load (w m²)

The production system adopted was extensive, with animals raised on native pastures dominated by hyperxerophilous caatinga vegetation, which is characterized by xeric plants, cacti, and shrubs. Supplementary concentrate feeding was provided once daily.

## Environmental variables

Air temperature and relative humidity were recorded using a digital thermo-hygrometer and black globe thermometer in the same setting and time as the morphophysiological measurements, yielding paired ambient data per animal. We computed Radiant Thermal Load (RTL) as per Silva (2000):

$$\:\mathrm{R}\mathrm{H}\mathrm{L}\hspace{0.17em}=\hspace{0.17em}1.053\:\times\:\:\mathrm{h}\mathrm{c}\:\times\:\:(\mathrm{T}\mathrm{G}\mathrm{N}\:\--\:\mathrm{T}\mathrm{a})\:+\sigma\:\mathrm{T}\mathrm{G}\mathrm{N}^4\:(\mathrm{W}/\mathrm{m}^2)$$

where:

$\:{h}_{c}\:$is the globe convective coefficient (W·m⁻²·K⁻¹),

$\:{T}_{BG}$the black globe temperature (K),

$\:{T}_{a}$the air temperature (K),

$\:\sigma\:$ Stefan–Boltzmann constant (5.6697 × 10⁻⁸ W/m²·K⁴).

Table 1 presents the characterization of the thermal environment for the experiments conducted in Spain and Brazil.

**Table 1:** Environmental conditions recorded during the trials in Spain and Brazil

Variables/Country		Mean	SEM	Minimum	Maximum
AT	Spain	23.52	0.26	12.52	39.80
	Brazil	34.63	0.15	30.08	42.90
RH	Spain	57.78	0.55	25.00	82.00
	Brazil	38.33	0.46	25.00	56.00
RHL	Spain	421.48	1.42	346.03	648.72
	Brazil	616.43	6.02	523.60	851.33

AT - air temperature (°C), RH – relative humidity (%), RHL - Radiant heat load (w m^-^²); SEM - Standard error of the mean

### Data collection

Data for the five breeds were collected in March and September. Physiological variables were recorded simultaneously with the meteorological variables; subsequently, morphological variables were measured.

### Thermoregulatory responses

Respiratory rate was measured by stethoscope auscultation over 1 min. Rectal temperature was obtained with a digital clinical thermometer (up to 44 °C), inserted ~ 5 cm into the rectum, in contact with the mucosa.

### Coat morphological traits

Coat samples were taken from the flank using duckbill pliers to determine mean hair length and mean hair diameter. Coat thickness was measured in situ at the same site using a millimetric metal ruler with slider, inserted perpendicular to the surface until contacting the skin; the slider was brought to the outer hair surface for reading.

Hair length was determined in the laboratory with a digital caliper by measuring the 10 longest fibers (visually selected) and computing the arithmetic mean (Udo 1978). Hair diameter was then measured on the same fibers with a digital micrometer, and the arithmetic mean was computed (Lee 1953).

### Statistical methods

Morphophysiological responses of the goat populations were analyzed separately, considering the climatic and environmental particularities of each country. Thus, this study aimed to characterize the adaptive profile and identify the main adaptive mechanisms of each population within its respective environmental context, following the approach used by Ribeiro et al. (2015), who determined the adaptive profile of Azul goats (Brazil) and Garfagnina goats (Italy) and interpreted population differences in light of environmental conditions.

Step 1 (*Univariate analyses*) The sample size was defined a priori based on a balance between statistical power, feasibility constraints, and ethical considerations related to animal experimentation. Previous studies assessing thermoregulatory and coat traits in ruminants under contrasting thermal environments have reported moderate to large effect sizes for breed differences in rectal temperature, respiratory rate, hair length, and coat thickness (e.g., Ribeiro et al. 2026; Silveira et al. 2024; Vasconcelos et al. 2021 ab). Assuming conservative medium effect sizes (Cohen’s *f* ≈ 0.25–0.30), an alpha level of 0.05, and a target statistical power of 0.80 for detecting between-breed differences in univariate analyses, an a priori power analysis indicated that sample sizes on the order of 25–30 animals per group would be sufficient. Accordingly, we evaluated 30 animals per breed, which is consistent with sample sizes commonly adopted in experimental and field studies on thermal adaptation in small ruminants. Residual normality was assessed with the Shapiro–Wilk test, homogeneity of variances with Levene’s test, and independence by design; all assumptions were met. A post hoc power analysis based on the observed effect sizes for the primary outcomes indicated ≥ 80% power at α = 0.05 with the current sample size. Differences in physiological and morphological traits among breeds, for each variable, were then tested using a repeated-measures ANOVA, with breed as a fixed between-subjects factor and time as a within-subjects factor. Multiple comparisons among breeds were performed with Tukey’s at α = 0.05. ANOVA results are reported on the original measurement scale.

Step 2 (*Standardization and suitability for multivariate analyses*). For all multivariate procedures, variables were standardized to z-scores, and analyses were based on correlation matrices. Within each breed, correlation structure was decomposed via principal component analysis (PCA) on the z-score correlation matrix, using varimax rotation to maximize loading simplicity and interpretability. The factor analysis model is expressed:

$$\matrix{{{X_1} = {a_{11}} \times \,{F_1} + \,{a_{12}} \times \,{F_2} + \cdots \, + \,{a_{1m}} \times \,{F_m} + \,{e_p}} \cr{{X_2} = {a_{21}} \times \,{F_2} + \,{a_{21}} \times \,{F_2} + \cdots \, + \,{a_{2m}} \times \,{F_m} + \,{e_p}} \cr\vdots \cr{{X_p} = {a_{p1}} \times \,{F_1} + \,{a_{p1}} \times \,{F_2} + \cdots \, + \,{a_{pm}} \times \,{F_m} + \,{e_p}} \cr}$$

In which:

$\:{X}_{p}$is the p ^th^ score of the standardized variable (*p* = 1, 2, …, m).

$\:{F}_{m}$is the extracted factor, $\:{a}_{pm}$is the factor loading, and $\:{e}_{p}$is the error.

Factor scores for each group were estimated by multiplying standardized variables by the coefficient of the corresponding factor score, as follows

$$\matrix{{{F_1} = {d_{11}} \times \,{X_1} + \,{d_{12}} \times \,{X_2} + \, \cdots \, + \,{d_{1j}} \times \,{X_{jp}}} \cr{\,\,{F_2} = {d_{21}} \times \,{X_2} + \,{d_{21}} \times \,{X_2} + \cdots \, + \,{d_{2j}} \times \,{X_{jp}}} \cr\vdots \cr{\,{F_j} = {d_{p1}} \times \,{X_1} + \,{a_{j1}} \times \,{X_{2\,}} + \, \cdots \, + \,{d_{jp}} \times \,{X_{jp}}} \cr}$$

In which:

$\:{F}_{j}$is the j- ^th^ factor extracted,

$\:{d}_{pj}$is the factor score coefficient

p is the number of variables.

Sampling adequacy was evaluated with KMO by population (Majorera goats = 0.58; Palmera goats = 0.62; North Tinerfeña goats = 0.50; Canindé goats = 0.58; Moxotó goats = 0.51) and Bartlett’s test of sphericity, which was significant in all panels (*P* < 0.001), supporting multivariate reduction/ordination. Component retention followed the Kaiser criterion (eigenvalue > 1) with scree-plot inspection; factor loadings |λ| ≥ 0.40 were considered salient. The percentages reported for the first two axes correspond to the variance explained in the rotated solution; biplots of the first two components were generated for each breed.

Step 3 (*Between-group structure*) Relationships among breeds were summarized using correspondence analysis applied to the breed × standardized-variable matrix, with centering and range standardization to equalize trait contributions. This approach described the gradient contrasting morphology-centered versus physiology-centered profiles and summarized the inertia explained by the leading axes.

Step 4 (*Discriminant analysis*) Group separation and classification performance were evaluated via canonical discriminant analysis with a pooled covariance matrix. The general CDA model is described:

$$\:{Z}_{n}=\:\propto\:+\:{\beta\:}_{1}{X}_{1}+{\beta\:}_{2}{X}_{2}+\:\cdots\:+\:{\beta\:}_{n}{X}_{n}\:\:$$

In which:

$\:{Z}_{n}$is the dependent variable (databases),

$\:\propto\:$is the intercept,

$\:{X}_{i}$are the explanatory variables,

$\:{\beta\:}_{i}$are the discriminant coefficients for each explanatory variable.

A stepwise variable-selection procedure based on Wilks’ lambda (entry/removal at *P* = 0.05/0.10) identified the most informative variables determining the animals’ adaptive profile. Prior probabilities were set proportional to breed sample sizes. Classification accuracy was estimated overall and by breed, and the between-group variance attributed to the first two canonical functions was reported. Robustness was assessed by leave-one-out cross-validation, and Box’s M was inspected; given the verified assumptions and observed performance, the linear solution was retained.

## Results

Results in Tables 2  and 3 indicate differences between Brazilian and Spanish breeds for the evaluated traits. Among the Spanish breeds (Majorera, Palmera, and North Tinerfeña), rectal temperature (RT) did not vary among breeds (*p* > 0.05), remaining around 39.2 °C. In contrast, respiratory rate (RR) differed among them (*p* < 0.05), with North Tinerfeña showing higher values (≈ 39.3 breaths/min) than Majorera and Palmera, which did not differ from each other (≈ 36–37 breaths/min). Coat architecture also varied among the Spanish breeds, with greater coat thickness (CT) and hair length (HL) in Palmera and North Tinerfeña (≈ 6.8–7.5 mm and ≈ 127 mm, respectively) compared with Majorera (≈ 1.06 mm and ≈ 36.7 mm; *p* < 0.05). Hair diameter (HD) also differed among the Spanish breeds (*p* < 0.05), being lowest in Majorera, and highest in North Tinerfeña.

**Table 2:** Thermal homeostasis parameters and coat architecture in autochthonous Spanish goat breeds (*n* = 90 goats)

Breeds	RT		RR		CT		HD		HL	
	Mean	SEM	Mean	SEM	Mean	SEM	Mean	SEM	Mean	SEM
Majorera	39.27^a^	0.02	36.10^b^	1.07	1.06^b^	0.03	0.04^c^	0.0006	36.74^b^	0.41
Palmera	39.27^a^	0.03	36.68^b^	0.88	7.53^a^	0.50	0.06^b^	0.0009	127.72^a^	3.57
North Tinerfeña	39.18^a^	0.03	39.33^a^	0.88	6.84^a^	0.29	0.07^a^	0.0005	127.63^a^	2.44

Note: RT - rectal temperature (°C); RR - respiratory rate (breaths/min); CT - coat thickness (mm); HL - hair length (mm); HD - hair diameter (µm). SEM - Standard error of the mean

^a–c^ Different letters within the same column indicate differences according to Tukey’s test

For the Brazilian breeds (Canindé and Moxotó; Table 3), RT did not differ between breeds (*p* > 0.05), with values around 39.45–39.50 °C. However, Canindé had a higher RR (*p* < 0.05), reaching approximately 42.1 breaths/min, whereas Moxotó showed about 38.3 breaths/min. CT also differed between Brazilian breeds (*p* < 0.05), being higher in Canindé (≈ 0.56 mm) than in Moxotó (≈ 0.38 mm). HD was similar between Canindé and Moxotó (*p* > 0.05), while HL was higher in Canindé (*p* < 0.05).

**Table 3:** Thermal homeostasis parameters and coat architecture in autochthonous Brazilian goat breeds (*n* = 60 goats)

Breeds	RT		RR		CT		HD		HL	
	Mean	SEM	Mean	SEM	Mean	SEM	Mean	SEM	Mean	SEM
Canindé	39.45^a^	0.07	42.13^a^	1.62	0.56^a^	0.01	0.06^a^	0.0039	26.76^a^	0.55
Moxotó	39.50^a^	0.05	38.34^b^	1.55	0.38^b^	0.01	0.05^a^	0.0011	24.09^b^	0.68

Note: RT - rectal temperature (°C); RR - respiratory rate (breaths/min); CT - coat thickness (mm); HL - hair length (mm); HD - hair diameter (µm). SEM - Standard error of the mean

^a–c^ Different letters within the same column indicate differences according to Tukey’s test

Figure 1 deepens this dichotomy by decomposing the correlation structure within each population. In Majorera (Axis 1 = 35.42%; Axis 2 = 15.10%),  morphological and physiological variables show partial separation, suggesting a moderate decoupling between coat characteristics and thermophysiological responses. . In Palmera (Axis 1 = 42.10%; Axis 2 = 20.61%), the main gradient is clearly morphological: HL and HD are opposed to RT, indicating that within-breed differences are explained primarily by the coat. In North Tinerfeña (Axis 1 = 34.78%; Axis 2 = 17.89%), a clear trade-off emerges: RT/RR load on the positive semi-axis of Axis 1, and HL projects on the negative side, such that greater insulation tends to reduce the need for physiological responses.In the Spanish goat populations, it was observed that an increase in ambient temperature is positively associated with an increase in respiratory rate.  Among the Brazilian breeds, the orientation is reversed. In Canindé (Axis 1 = 29.82%; Axis 2 = 23.81%) and Moxotó (Axis 1 = 25.86%; Axis 2 = 24.90%), there is a strong association between RT and RR, indicating that within-breed variability is governed chiefly by physiological components.

**Fig. 2:**
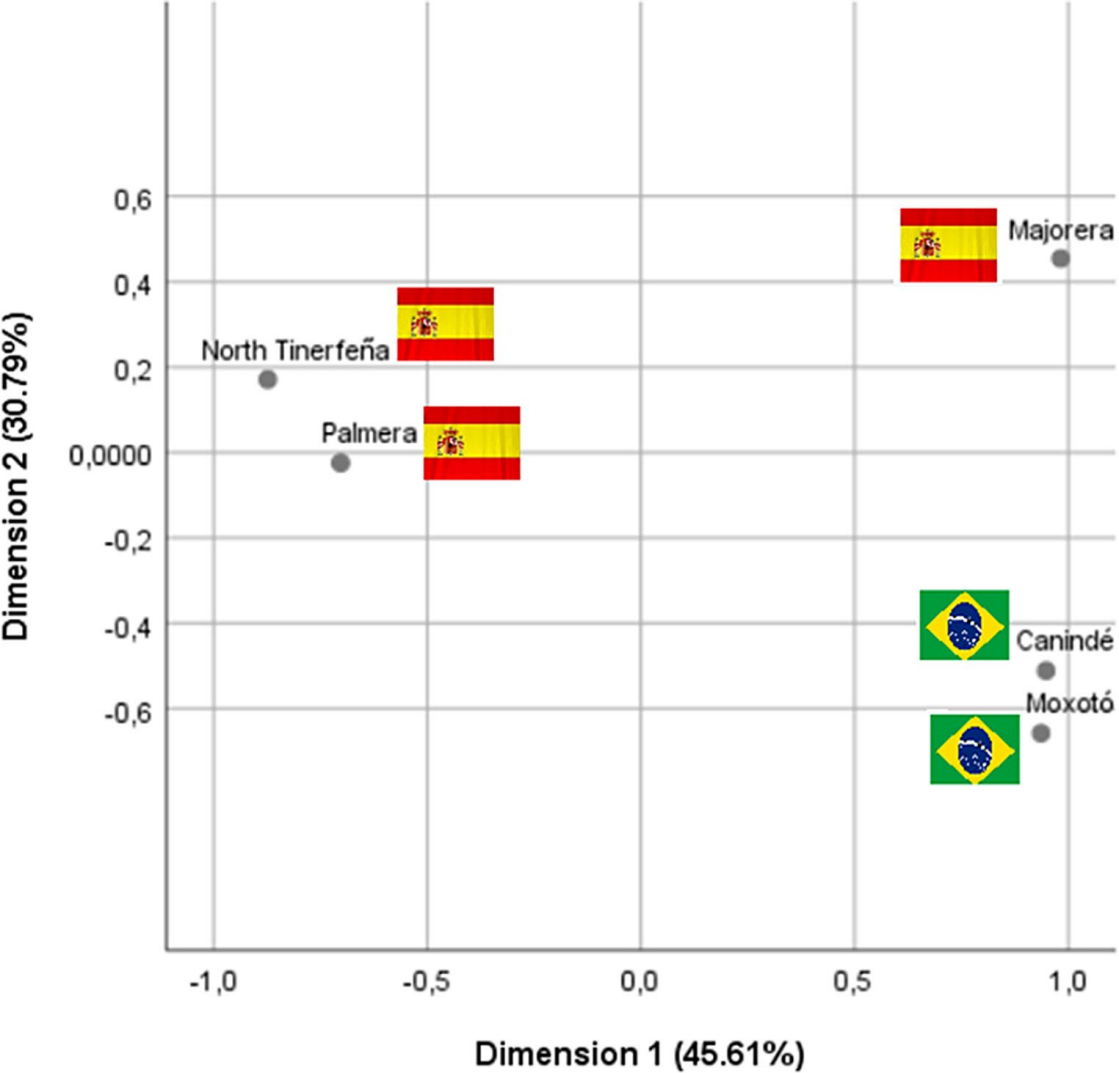
Correspondence analysis of the five goat breeds based on morphological and physiological variables

The correspondence analysis (Fig. 2) summarized between-group differences and clearly separated the Brazilian and Spanish breeds along Axis 1 (45.61%), with Majorera displaced toward the upper quadrant and Palmera/North Tinerfeña concentrated on the negative semi-axis, whereas Canindé and Moxotó positioned at the positive . Axis 2 (30.79%) refined within-block distinctions, reinforcing the morphology-centered (Spain) versus physiology-centered (Brazil) opposition.

**Fig. 3:**
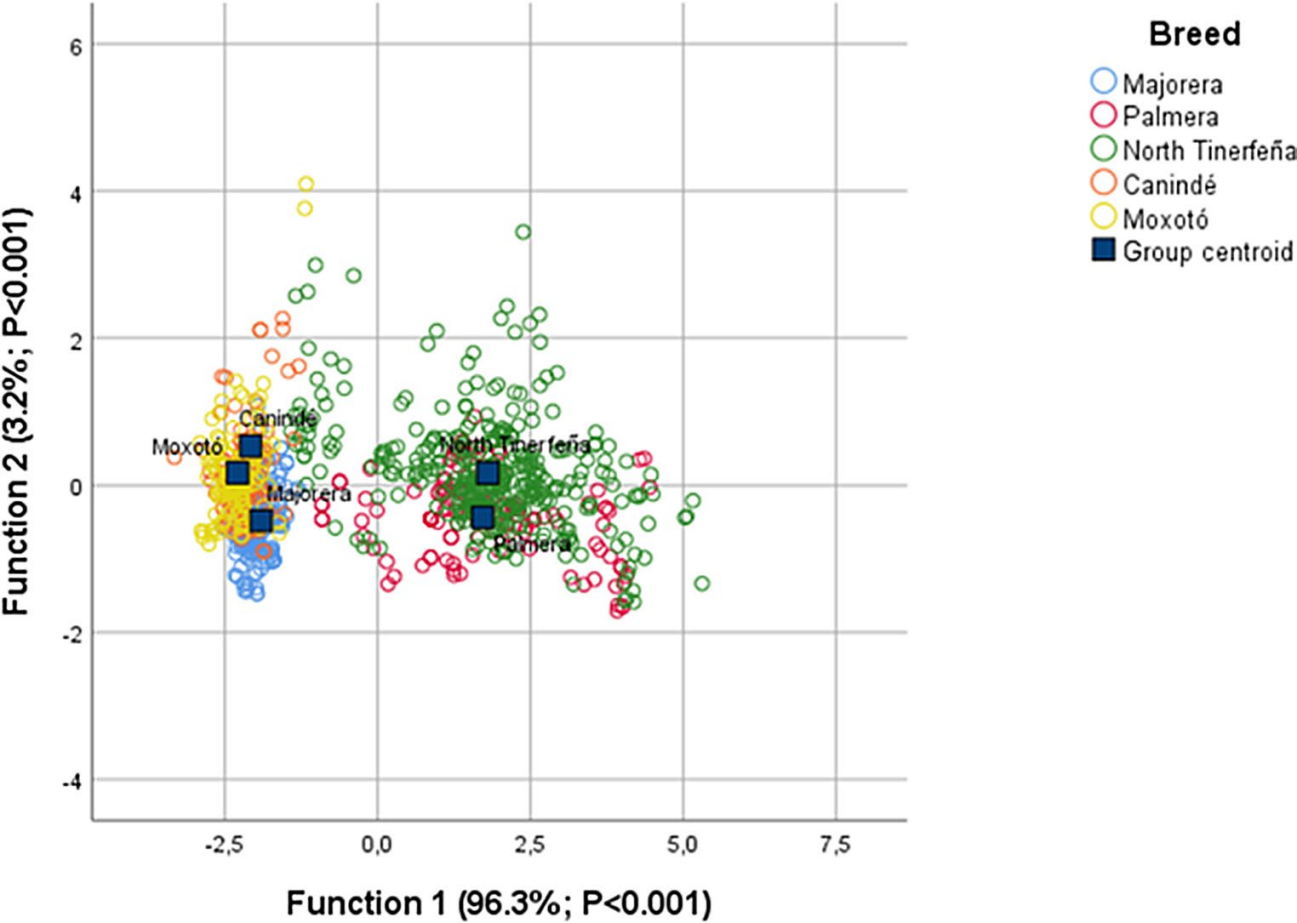
Discriminant analysis of the five goat breeds based on morphological and physiological variables

Discriminant analysis corroborated the separation (Wilks’ lambda *P* < 0.001) and quantified the classificatory capacity of the variables (Table 4; Fig. 3). Considering physiological indicators alone, overall accuracy was 38.5%, with notably high performance for North Tinerfeña (94.7%) and low for the others. With morphological variables, global accuracy rose to 58.4%, driven by high correct-classification rates in Majorera (96.7%) and North Tinerfeña (90%), while Canindé and Moxotó remained modest (≈ 16.7% and 9.2%). When all indicators were combined, total accuracy reached 60.6%, with 92.8% of Majorera individuals and 90% of North Tinerfeña correctly allocated. The Brazilian breeds retained lower accuracies (≈ 21.7% in Canindé and 22.7% in Moxotó). The first two canonical functions explained virtually all between-group variance (Function 1 = 96.3%; Function 2 = 3.2%; *p* < 0.001), with hair length and coat thickness as the main adaptive descriptors. The canonical plot (Fig. 3) mirrors these results: centroids summarize two adaptive profiles, with Majorera isolated on the left and forming a compact cloud together with Canindé and Moxotó, and Palmera and North Tinerfeña partially overlapping in the central region. The geometry of the individual clouds further shows that separation is governed mostly by Function 1, consistent with the weight of HL/CT in interbreed discrimination and with the structural opposition between morphological insulation and physiological adjustment observed in the biplots.

**Table 4:** Discriminant analysis of physiological and morphological profiles in five goat breeds: classification accuracy by breed, explained variance, and Wilks’ Lambda

Indicators	C_CC_^1^ (%)	Classification^2^ (%)					Variance Explained (%)		Lambda of Wilks^3^ (*P* – value)		Main Traits^4^
		Majorera	Palmera	North Tinerfeña	Canindé	Moxotó	F_1_	F_2_	F_1_	F_2_	
Physiological	38.5	0	0	94.7	0	6.7	100		< 0.001	-	RT
Morphological	58.4	96.7	0	90	16.7	9.2	97	2.9	< 0.001	< 0.001	HL < CT
All traits	60.6	92.8	4	90	21.7	22.70	96.3	3.2	< 0.001	< 0.001	HL < CT

^1^ Total percentage of cases correctly classified: C_CC_= cases correctly classified

^2^ Percentage of cases correctly classified by breed

^3^ Statistic test: Canonical functions (F_1_ and F_2_) with *P* < 0.05 of Wilks’ Lambda were considered significant

^4^ Main traits of the groups: RT - rectal temperature (°C); CT - coat thickness (mm); HL - hair length (mm)

## Discussion

The results clearly delineated two contrasting adaptive profiles among the goat breeds evaluated: a profile of morphological insulation, characterized by long, dense hair with fine fiber diameter; and a profile of physiological adjustment, marked by short hair and amplified thermoregulatory responses, with elevated rectal temperatures and respiratory rates. These findings align with mechanisms already described for small ruminants in hot climates: morphological traits reduce environmental heat gain; physiological responses such as increased respiratory rate, sweating, reduced basal metabolism, and endocrine adjustments dissipate excess internal heat (Berihulay et al. 2019; ; Silveira et al. 2024). In other words, the thermal environment modulates caprine morphophysiological responses, since animals can withstand heat stress either by physically isolating heat entry through hair morphology, as in Spanish goats better adapted to cooler climates, or by increasing metabolic heat loss, as in Brazilian goats raised under high temperatures. The populations studied here exemplify these two adaptive pathways with clarity.

In the Spanish breeds Palmera and North Tinerfeña, the strategy of morphological insulation predominated. The long and thick hair observed, with hair lengths of approximately 127 mm and coat thickness of approximately 7 mm, acts as an insulating barrier that limits heat load on the body surface. This insulation operated similarly to what is observed in coarse-wool sheep or goats from arid climates, since it creates a trapped air layer that shields the skin from direct solar radiation (da Silva et al. 2025a, b a; Silveira et al. 2024). Classic studies indicate that long-haired goats tolerate intense insolation better than short-haired goats, exhibiting smaller increases in body temperature and respiratory rate under radiant heat. For example, Acharya et al. (1995) reported that long-haired goats maintain rectal temperature and physiological parameters more stably during five days of continuous sun exposure, whereas short-haired individuals show respiratory acceleration and larger thermal oscillations, which indicates lower heat tolerance. In the present study, this pattern was reflected in the Spanish breeds, which recorded relatively low respiratory rates, approximately 36 breaths per minute even under warm conditions, which suggests a reduced need for active heat loss due to the protection afforded by the coat.

It is important to note, however, that excessive insulation can become a burden in very hot or humid climates. Our data indicate that the Palmera breed, which bears the thickest coat, nevertheless showed a slight thermal rise and likely required greater cutaneous evaporation in summer. This echoes recent findings in the Canary Islands, in which Palmera, with longer and denser hair, proved more susceptible to heat stress than Majorera under the same climatic conditions (da Silva et al. 2025a, b). In that context, Palmera goats had to mobilize more evaporative mechanisms to offset overheating, whereas Majorera goats, with naturally shorter hair, dissipated heat more efficiently and maintained superior milk production. In sum, although abundant hair is advantageous for mitigating solar radiation and daily thermal oscillations, there are trade-offs; a fine balance between insulation and dissipation is required, depending on the environmental context.

By contrast, the Brazilian breeds Canindé and Moxotó displayed the opposite profile, centered on physiological adjustments to maintain homeothermy. The short, sparse hair with coarser fibers observed in these breeds, with lengths of 24 to 27 mm and a thin coat, allows faster environmental heat gain; however, these animals compensate by intensively activating mechanisms of metabolic heat loss, notably accelerated pulmonary ventilation. Our results showed that Canindé and Moxotó reached the highest respiratory rates, 38 to 42 breaths per minute, and rectal temperatures near 39.5 °C during heat stress, which evidences reliance on evaporative cooling through panting or polypnea to dissipate heat. This behavior accords with the general thermoregulatory pattern of goats: under high temperatures, the first response is a marked increase in respiratory rate, followed by increased cutaneous sweating, to expel excess body heat (Maia et al. 2016; El-Sherbiny et al. 2023). Goats are efficient homeotherms in warm environments, since they use the lungs and the skin as the main avenues for heat loss (Vasconcelos et al. 2021; Lima et al. 2022). In such cases, the absence of robust insulation renders physiological mechanisms even more crucial. Furthermore, traits typical of breeds adapted to humid and semiarid tropics in goats and ruminants in general, such as short and sleek hair and smaller body size, favor heat dissipation (Farias et al. 2024). For example, West African Dwarf goats, native to hot-humid climates, have short, fine, and sleek hair that facilitates heat loss and are often associated with high heat-stress tolerance in these conditions (Daramola et al. 2009). By analogy, Canindé and Moxotó appear to have evolved morphologically to favor dissipation via high respiratory rates and possible sweating, as demonstrated for Canindé goats raised under similar conditions to those of this study (Façanha et al. 2020). Although energetically costly, since it entails greater respiratory work and risk of respiratory alkalosis, this strategy remains effective for keeping body temperature within safe limits when morphological protection is limited (Lima et al. 2022).

Canonical discriminant analysis highlighted rectal temperature as the physiological trait with the greatest discriminant power among breeds, a classic indicator of the ability to tolerate excessive heat (Ferreira et al. 2020). The Brazilian breeds reached rectal temperature values significantly higher than those of the Spanish breeds, which signals greater internal heat accumulation before reaching equilibrium. This higher threshold may reflect an adaptation to store heat temporarily during the daytime peak and release it when conditions ease, a strategy observed in desert species that minimizes water losses through evaporation. Another hypothesis is that breeds adapted to high temperatures present a higher basal rectal temperature as an adaptive mechanism. This is consistent with projections to 2100 suggesting that ruminants raised in the tropics may elevate rectal temperature as an adaptive response to progressive warming (Silveira et al. 2026).

Overall, the dichotomy between morphological insulation in Spanish populations and physiological adjustment in Brazilian populations observed in the five breeds analyzed illustrates an adaptive gradient shaped by distinct environmental pressures. Breeds originating from semiarid or open tropical environments, such as Moxotó and Canindé in Northeastern Brazil, seem to have been selected to maximize active heat dissipation, since they adopt a light coat that facilitates heat exchange and tolerate broad physiological variation, including intense panting and daily fluctuations in body temperature. In turn, breeds developed in insular subtropical and montane climates, such as Palmera and North Tinerfeña in the Canary Islands, prioritized a morphological barrier to heat, with a thick coat, often lighter in color, that reflects and blocks much of the incident solar radiation, which reduces the need for drastic physiological responses (da Silva et al. 2025a, b ). This dual adaptive architecture explains the differences observed in our data, with opposing ordination axes in the biplots, clear separation in correspondence diagrams and in discriminant functions, and it finds parallels across several domesticated ruminants. For example, when comparing the adaptive profiles of Azul goats from Brazil and Garfagnina goats from Italy, researchers reported that short hair and larger hair diameter in the Azul population favored air renewal near the skin; this population responded more rapidly to environmental changes through physiological adjustments and better use of adaptive mechanisms, even under continuous heat stress, compared with Garfagnina (Ribeiro et al. 2016).

The multivariate proximity observed between the Majorera breed and the Brazilian breeds in canonical space and ordination analyses represents a central finding of this study, rather than merely an incidental observation. Although Majorera is a breed originating from the Canary Islands, its closer positioning to the Brazilian groups suggests that it occupies a functionally intermediate position along a continuous spectrum of adaptive strategies, approaching a profile more reliant on physiological adjustments than that observed for Palmera and North Tinerfeña. This finding reinforces the interpretation that thermal adaptation in goats is not organized as a strict dichotomy between “morphology” versus “physiology,” but rather as an adaptive continuum, in which different breeds combine, in varying proportions, mechanisms of morphological insulation and physiological heat dissipation. From an evolutionary and functional perspective, Majorera may represent a transitional adaptive phenotype, possibly shaped by historically more arid and open environments within the Canary archipelago, which makes it functionally convergent with native breeds from the Brazilian semi-arid region. This functional convergence has direct implications for conservation and breeding programs, as it indicates that breeds from distinct geographical origins may share similar adaptive solutions in response to convergent thermal pressures (Silveira et al., 2024).

A particularly notable result was the low classification accuracy for the Palmera breed when physiological or morphological variables were considered separately, with accuracy close to zero in both cases. This pattern suggests that Palmera exhibits an intermediate adaptive profile that largely overlaps with that of the North Tinerfeña, leading to frequent misclassifications due to the phenotypic and functional similarity between these two breeds, which share a predominantly morphology-based adaptive strategy. As a result, the classifier tends to confuse Palmera individuals with North Tinerfeña, drastically reducing discriminative power when only a restricted set of variables is considered. Alternatively, this finding may also indicate that the set of variables evaluated (rectal temperature, respiratory rate, and basic coat traits), although informative for distinguishing adaptive extremes, is insufficient to capture nuances specific to Palmera’s adaptive strategy. It is plausible that other relevant components of thermotolerance—such as sweating rate, cutaneous conductance, surface temperature, sweat gland characteristics, shade-seeking behavior, and endocrine variables—play a more determinant role in this breed, blurring its separation when only the indicators used here are considered.

Palmera and North Tinerfeña populations, due to their thick coats, tend to show a smaller increase in respiratory rate during heat waves, although they may experience a greater decline in milk yield if dissipation is insufficient (da Silva et al. 2025a, b ). In our study, the high classification accuracy for Spanish breeds using coat variables alone, 90 to 97% for North Tinerfeña and Majorera, reinforces that their morphological attributes are distinctive and decisive for discriminating adaptive profiles in production animals. A similar result was reported in sheep, in which skin and coat traits such as hair length, coat reflectance, the proportion of epidermal area occupied by sweat glands, and skin thickness were the most important for separating breeds with different coat colors in Central Brazil (Castanheira et al. 2010). Façanha et al. (2019) verified that hair diameter, length, thickness, and density classified Brahman bulls according to the seasons in the Brazilian Cerrado with high accuracy for their group of origin, and Baena et al. (2019) indicated that hair number and coat length are suitable parameters for assessing taurine cattle adaptation to heat.

On the other hand, the lower capacity of isolated physiological variables to distinguish Brazilian breeds, with accuracy below 40%, indicates that responses such as panting and hyperthermia are widely shared by these populations under heat stress, therefore they are not exclusive to one breed or another, which suggests convergent adaptation. Ultimately, although all breeds evaluated can survive in hot climates, the evolutionary pathways taken to cope with heat differ. Recognizing these differences is crucial for management and breeding strategies. Crossbreeding or selection programs that introduce genetics from morphologically insulating breeds into extremely hot environments may require additional care; physiologically adjusting breeds rely heavily on water access and on practices that minimize evaporative stress. In sum, the diversity of adaptive profiles described here reflects the ability of goats to balance morphological insulation and physiological dissipation in the face of heat; understanding this balance enables better use of each breed’s productive potential within its thermal comfort limits.

Despite the robustness of the results and the consistency of the observed patterns, some limitations should be acknowledged. First, there is a potential confounding between environmental and management effects, even though univariate comparisons were conducted separately by context, given that production systems differed between the evaluated settings (intensive in the Canary Islands and semi-intensive/extensive in the Brazilian semi-arid region). Thus, part of the morphophysiological differences observed among populations may reflect not only genetic or phenotypic adaptation to the thermal environment, but also responses shaped by management practices, feeding regimes, and local microenvironments. Second, measurements were collected at only two time points over the year, which limits the capture of finer seasonal variability and longer-term adaptive responses to extreme thermal events, such as prolonged heat waves. Third, the study focused exclusively on adult, non-pregnant, non-lactating females, which constrains the generalizability of the findings to males, juveniles, or lactating females, categories that exhibit distinct metabolic demands and thermoregulatory responses. Finally, the physiological assessment relied on only two core variables (rectal temperature and respiratory rate), which, although widely validated as indicators of heat stress, do not fully capture the complexity of thermoregulatory processes, which also involve parameters such as sweating rate, skin or surface temperature, hematological and hormonal variables, and metabolic markers. Acknowledging these limitations does not undermine the findings, but rather highlights the need for future studies integrating more controlled experimental designs, higher temporal resolution, and a broader set of physiological indicators to deepen the understanding of adaptive profiles across contrasting climatic contexts.

## Conclusion

This study shows that heat tolerance in goats can arise through distinct yet complementary pathways: morphological insulation, with long, thick, coarse fibers that attenuate radiative heat gain and reduce the need for hyperventilation; and physiological adjustment, with short, thin, fine hair combined with marked increases in breathing and mild thermal oscillations, which favors evaporative heat loss during thermal peaks. Multivariate analyses corroborated this dichotomy by showing that, in the Canary Islands breeds, especially Palmera and North Tinerfeña, coat morphology structures the main gradient of variation, whereas in the Brazilian breeds physiological components predominate. Group discrimination was more accurate when coat traits were considered, underscoring the predictive value of morphology for identifying adaptive profiles.

In applied terms, the results support conservation strategies that preserve intra- and interbreed diversity and guide differentiated breeding programs: in environments with high radiant load and limited water, prioritize genotypes with efficient morphological insulation; in contexts of persistent heat and management with water access, value genotypes with high physiological heat-dissipation capacity. By integrating morphology and physiology, this study offers a framework for selection aimed at thermal resilience and productive sustainability in the face of climate warming.

The findings presented here also open clear avenues for applied research on goat adaptation to climate warming. Future studies integrating genomic and functional genomics approaches with these morphological and physiological profiles could identify candidate genes and genomic regions associated with heat tolerance, providing objective bases for marker-assisted and genomics-informed selection programs.

## Data Availability

The data that support the findings of this study are available from the corresponding author upon reasonable request.

## Code availability

No applicable.
